# A quantitative study on female sex workers’ mental health in Germany

**DOI:** 10.3389/fpubh.2025.1590151

**Published:** 2025-08-29

**Authors:** Franziska Kroehn-Liedtke, Olivia Kalinowski, Gizem Kaya, Anastasiia Lotysh, Hristiana Mihaylova, Krisztina Sipos, Annika Strunk, Lena Zerbe, Wulf Rössler, Meryam Schouler-Ocak

**Affiliations:** Department of Psychiatry and Neurosciences, Psychiatric University Clinic of Charité at St. Hedwig Hospital, Berlin, Germany

**Keywords:** mental health, sex work, prevalence of mental disorders, risk factors, social workers

## Abstract

**Introduction:**

Sex workers provide sexual services in exchange for monetary compensation, encompassing a range of activities such as escorting, pornography and street-based or online sex work. Individuals doing sex work are frequently referred to as a vulnerable group when it comes to their mental health. Despite the legality of sex work in Germany, there are significant risk factors associated with negative impact on the mental health, such as stigmatization, precarious working conditions and experienced violence. The aim of this study was to examine specific sex work settings. It was hypothesized that sex workers report high levels of structural stress and psychological distress and that specific work-related factors (e.g., stigma, safety concerns, interactions with clients) would significantly predict mental health outcomes.

**Methods:**

Since the majority of sex workers is female, the mental health of 403 women engaged in sex work was assessed in this study using quantitative interviews with standardized instruments. To acquire prevalence rates of mental disorders and compare them with others engaged in high-stress environments a control group of 157 female social workers was interviewed. To investigate the hypotheses descriptive analyses, Chi^2^-tests, logistic regression and cluster analysis were used. This study employs a cross-sectional design to equally reflect the different characteristics of the heterogeneous groups such as age, educational level, background and different work settings.

**Results:**

As expected, mental disorder prevalences were higher among sex workers than in the general population and in the control group. However, logistic regression identified risk factors for mental illness, including residence status (OR = 1.33, CI = 1.05–1.69, *p* = 0.018; Ref = no), homelessness (OR = 0.11, CI = 0.02–0.48, *p* = 0.003; Ref = yes), income status (OR = 0.50, CI = 0.25–0.97, *p* = 0.042; Ref = low), perceived threat or control (OR = 0.15, CI = 0.03–0.84, *p* = 0.030; Ref = no) and work-related stress (OR = 2.83, CI = 1.11–7.18, *p* = 0.029; Ref = low) emerging psychological and structural stressors as crucial factors.

**Discussion:**

These findings highlight the need for special health care services and improved economic and legal security of sex workers. Policy changes focusing on improved medical and psychological care and structural interventions aiming economic and social needs while fostering societal acceptance and understanding are required.

## Introduction

Sex work, often referred to as prostitution, remains a controversial topic and is subject to frequent prejudices about its risk for the mental health of those involved (1, 2). While the term sex work encompasses a range of sexual services like for example escorting, stripping or webcam modeling, pornography and bondage, discipline, dominance/submission and sadism/masochism services (BDSM), prostitution often refers to the exchange of sexual intercourse for monetary compensation. The latter term is frequently used in a legalistic context and, despite its widespread prevalence, continues to be associated with societal stigmatization (3–7). While sex work is legal in Germany, it is somewhat intertwined with criminal issues such as human trafficking, forced prostitution and organized crime. The distinction between voluntary and coerced sex work is often difficult to determine, complicating both legal proceedings and support mechanisms. Victims of trafficking may not seek help due to fear of retaliation, lack of legal residence, or the inability to identify themselves as victims. As a result, these criminal dimensions are central for understanding the full social and legal context of sex work beyond its formal legal status (8). To further understand the vulnerabilities associated with sex work, it is important to consider an intersectional framework. While sex workers face general societal stigmatization, this stigma is often compounded by overlapping systems of oppression such as sexism, racism and classism (9). Many sex workers in Germany are females from migrant backgrounds (9–12). Their experiences are shaped by legal status, social status of sex work, gender, ethnicity and socioeconomic status. These intersecting forms of marginalization contribute to structural barriers, including restricted access to healthcare, legal protection, and social services (7, 9). The legal status of sex work in Germany is shaped by two key laws: the Prostitution Act (ProstG, 2002), which formally legalized sex work and granted sex workers access to labor rights and social security and the Prostitute Protection Act (ProstSchG, 2017), which introduced mandatory registration and annual health consultations. However, this legal recognition exists alongside strict criminal provisions targeting human trafficking, forced prostitution and exploitation. This structure reflects a broader European pattern of ambiguity – while some countries like Germany and the Netherlands regulate sex work as legal labor, others such as Sweden and France follow the “Nordic model,” which criminalizes the purchase but not the selling of sex services (7–9).

According to the Federal Statistical Office, in 2024 there were 30,636 registered sex workers in Germany; however, estimates suggest the actual number of people involved in sex work is considerably higher (12). Women constitute the majority of sex workers, with estimates ranging from 70 to 90%, and a significant proportion – also 70 to 90% – report a history of migration (8, 10–12). Among the registered sex workers, a significant proportion are non-German nationals, with the most frequent nationalities being Romanian (36%), Bulgarian (11%), and Spanish (7%) (12). Despite the introduction of the Prostitute Protection Act (ProstSchG) in Germany in 2017, which mandates annual health consultations, the health status and medical care of individuals engaged in sex work remain inadequately addressed (8). Previous research has predominantly focused on sexually transmitted infections (STIs), such as Human Immunodeficiency Virus (HIV), emphasizing risks that affect both sex workers and their clients (5, 13, 14). There are few studies addressing mental health reporting elevated rates of mental disorders among sex workers. However, a more comprehensive and nuanced understanding of the underlying mechanisms, contextual factors, and protective resources remains limited (15, 16).

Although legalized under the Prostitution Act (ProstG) of 2002, sex work in Germany is regulated by policies that create barriers to accessing medical care, social counseling, legal protection and recourse in cases of discrimination (8, 9, 17). While the German model is paradigmatic for the legalization of sex work, certain aspects may render it illicit, such as working without the necessary permit, being coerced into providing sexual services, or being underage. An important factor influencing mental health in this context is the experience of violence, including childhood maltreatment and abuse, as well as violence encountered in the work environment (14, 18, 19). Violence is recognized as a significant risk factor for mental health issues, particularly among individuals with pre-existing vulnerabilities. Rössler et al. identified violence within the context of sex work as a critical factor contributing to mental health vulnerabilities, describing a “vicious cycle” in which mental health issues increase the risk of victimization, thereby exacerbating psychological stress (20, 21).

Existing literature has mainly focused on prevalence rates highlighting the elevated prevalence of mental disorders among sex workers (2, 20, 21). A study in Zurich found that sex workers experienced mental disorders at significantly higher rates than women in the general population (21). Annual prevalence rates for affective disorders were reported at 30.1%, approximately six times higher than the general population (5.6%), while anxiety disorders affected 33.7% of sex workers compared to 8.7% in the general population (21). In Germany, the annual prevalence of mental disorders in the adult general population is 27.8%, with anxiety disorders (15.4%), affective disorders (9.8%), and substance-related disorders (5.7%) being the most common (22). Unipolar depression alone accounts for 8.2% (22). Regarding the lifetime prevalence of various mental disorders in the German general population, data vary depending on the study and methodology. Substance-related disorders are the most common, with a lifetime prevalence of 25.8%, lifetime prevalence of affective disorders (including depression) is 12.3%. Anxiety disorders affect approximately 15.1% of the population over the course of their lifetime and estimates for PTSD vary between 1.5 and 2% (23).

The findings suggest that mental health impairments could not only be a predisposing factor for entering sex work but also a consequence of the stress associated with it (21). However, causal pathways are hard to investigate because they require specific study designs, such as longitudinal studies, which are difficult to employ in this specific study population due to several reasons, such as high mobility and frequent sociodemographic changes within this group. Conditions of sex work, such as employment in escort services, brothels or street prostitution, alongside factors related to migration status, play a significant role in determining the frequency and severity of mental health issues (24).

A systematic review conducted in 2023 further corroborated the high prevalence of mental health disorders among sex workers, reporting rates between 50 and 71%. Affective disorders were the most common, with prevalence rates ranging from 30 to 53.5%, including depression (24–61.5%), dysthymia (11.9%), and bipolar disorders (46.9%). Anxiety disorders were also prevalent, with rates between 13.6 and 51%, encompassing panic disorder (8.8%), social phobia (7.3%), and generalized anxiety disorder (5.2–8%). Post-traumatic stress disorder (PTSD) was diagnosed in 10–39.6% of the cases, frequently accompanied by dissociative symptoms such as depersonalization (50%), derealization (59%), flashbacks (65%), memory disturbances (68%), and emotional distancing (71%) (2).

A recently published systematic review and meta-analysis found substantial variability in prevalence rates of mental disorders such as anxiety, depression, PTSD, substance use and dependence emphasizing that prevalences are mediated by population subgroups (e.g., street prostitution or sex workers living with HIV), and depend on individual circumstances rather than on a fixed effect through sex work itself (25).

These findings underscore the critical need for comprehensive mental health care interventions tailored to sex workers’ unique needs, i.e., addressing occupational hazards related to sex work, improving access to social support and mitigating the impact of violence and discrimination. As previous literature primarily focuses on prevalence rates, comprehensive analyses examining the interplay of structural and psychological stressors that impact the mental health of sex workers are still lacking.

The aim of this study is to investigate risk factors for mental disorders in this population in comparison with a control group also engaged in emotionally demanding interpersonal interactions, in contexts involving trauma or high-stress environments. Since social workers often represent the support systems sex workers may engage with, e.g., outreach, mental health care or advocacy, this study seeks to compare the distribution of risk and protective factors for mental health in the two contrasting populations. Understanding mental health in both groups may offer insight into parallel or contrasting coping mechanisms, support needs, and burnout factors that inform targeted interventions in both fields.

### Aims of the study

Since the legalization of sex work in Germany through the Prostitution Act in 2002, a legal framework has existed that includes the right to medical care, social security and employment law aspects (26). Unlike in countries where sex work is criminalized, this creates a unique framework for assessing the mental health of sex workers in a regulated environment where their rights are legally recognized.

Female sex workers (FSW) in Berlin include both native German women and migrants from Eastern Europe, Asia and Africa (27, 28). The mental health challenges faced by these different groups can vary depending on cultural, economic and social factors (29). This diversity makes Berlin an important place to explore this intersectionality and since the city has various social services and programs specifically tailored to sex workers, offering counseling, health services and legal assistance, assessing mental health in this context allows to examine the impact of these facilities (28). Since sex work includes online work formats, considering this form of location-independent work is important, as different stressors and resources can also vary. Hence, the study seeks to compare the prevalence of mental disorders among adult FSW working in Germany with a control group of social workers and to examine the relationship between mental health outcomes and covariates such as structural factors (e.g., working conditions, economic and residential aspects) and psychological aspects (e.g., stigmatization).

Female social workers were chosen as a control group due to similarities in emotionally demanding labor, gendered occupational contexts and exposure to stressful interpersonal dynamics. Both groups mostly consist of women working in traditionally feminized roles, often under conditions of undervaluation, stigma and institutional neglect. Rather than comparing sex workers to a general population sample that may differ substantially in education, income, and working conditions, this comparison allows for a more precise examination of how sex work-specific stressors relate to mental health, beyond structural disadvantage alone.

The aim of this study is therefore to investigate the mental health of female sex workers in Berlin and throughout Germany across various sex work settings and working conditions, including location-independent online sex work. The study seeks to evaluate what psychological distress and symptoms occur among this population, what risk and resilience factors can be identified and how do these aspects differ from a comparable professional group outside of sex work?

## Materials and methods

### Participants

For sampling a non-proportional quota-sampling approach was chosen. This method defines key population characteristics as sampling categories. Although the size of these categories may not reflect their actual proportions in the population, this approach ensures adequate representation of smaller groups. The selected sampling categories identified as potential risk factors for poor mental health were “homelessness,” “unsteady monthly income,” “residence status,” “perceived job stress” and “experiences of threat and control.” For legal and ethical reasons, FSW under the age of 18 were excluded from the study, other exclusion criteria included refusal to participate, severe cognitive impairment, psychotic decompensation, and acute suicidality. To maximize participation, FSW were approached directly at various locations, such as outdoor areas, studios, brothels, and through escort services, as well as online through social media like Instagram. Study information was also distributed at these venues to encourage engagement. Recruitment was further supported by information and assistance centers for sex workers, as well as organizations caring for the homeless. Since online sex work is not limited to a specific location, FSW outside Berlin who offer services online were also included. As control group that consists mostly women who are similar in key demographic variables (e.g., age, work with clients, stressful working conditions, socio-economic status etc.) female social workers were chosen. The control group of social workers was contacted online via counseling networks throughout Germany and via personal connections.

### Procedure

To obtain detailed, contextualized accounts of experiences with mental distress, interviews were chosen for data collection. The interviews were conducted between June 2022 and November 2024. Data collection included sociodemographic data (age, gender, nationality, residence status, relationship, children, education etc.) and contextual factors (location and setting of work, number of working days and customers per week, monthly income, exposure to violence etc.). To investigate whether the mental health issues faced by sex workers are a direct result of their work or whether they are due to other socioeconomic or personal factors, and to ensure that the results are not limited to FSW but can potentially influence broader mental health policies or practices, a control group was interviewed as well. All interviews were conducted by two trained clinical psychologists or student assistants from the fields of medicine, psychology or public health. The total duration of the face-to-face interview ranged from 60 to 90 min. At all stages of communication, it was emphasized that interviews would be conducted in several languages, including German, English, Russian, Bulgarian, Ukrainian, Polish, Romanian, Turkish, Vietnamese and Hungarian. Participation required informed written consent, and participants were compensated with a fixed amount of 50 EUR for their time and expenses. The study received approval from the ethics commission of Charité – Universitätsmedizin Berlin (EA2/133/18).

### Instruments

Sociodemographic data and contextual variables were collected by the interviewers. Some of the answer options were predefined (e.g., what is your gender: female, male, diverse or regarding the approximate monthly income: no income, up to 1,000 or over 1,000) or open questions (e.g., what nationalities do you have?). Following the sociodemographic interview, participants independently completed a series of standardized questionnaires while the interviewer was present to assist. The short version of the SF-36 Health Questionnaire (SF-12) was used to assess general health status (30). This questionnaire measures health-related quality of life in eight domains: physical functioning, physical role, bodily pain, general health, vitality, social functioning, emotional role, and mental health. The response scale is Likert-type varying across items with 3-point scales (e.g., “yes, limited a lot,” “yes, limited a little,” “no, not limited at all”), 5-point scales (e.g., “all of the time” to “none of the time”) and 6-point scales (e.g., “excellent” to “poor”). It demonstrates good reliability (Cronbach’s alpha > 0.80 for summary scores), good construct validity, and sensitivity to changes in health over time. The Perceived and Anticipated Stigma Scale (PASS-24) was used to assess experiences and expectations of (self-)stigma (31). It consists of 24 items covering the three dimensions of perceived, experienced, and anticipated stigma. The response scale is a 5-point Likert scale ranging from 0 = “strongly disagree” to 4 = “strongly agree.” It demonstrates high internal consistency (Cronbach’s *α* > 0.85 across all subscales) and good convergent validity with related constructs (e.g., self-esteem, social support). To assess traumatic childhood experiences, the short version of the Childhood Trauma Questionnaire (CTQ-SF) was used, using five subscales: emotional, physical, and sexual abuse, and emotional and physical neglect (32). The response scale is a 5-point Likert scale ranging from 1 = “never true” to 5 = “very often true.” It includes a three-item minimization/denial scale to detect underreporting and demonstrates excellent internal consistency (*α* > 0.90 for the total score; *α* > 0.70 for the subscales) as well as high convergent and discriminant validity. Coping strategies were assessed with the Brief COPE Inventory (Coping Orientation to Problems Experienced) using 14 subscales, each with two items, measuring active coping, denial, substance use, seeking emotional support, behavioral disengagement, positive reframing, etc. (33). The response scale is a 4-point Likert scale ranging from 1 = “I have not been doing this at all” to 4 = “I’ve been doing this a lot.” The inventory shows acceptable internal consistency for most subscales (*α* = 0.50–0.90) and good construct and criterion validity. The Big Five personality dimensions of extraversion, agreeableness, conscientiousness, emotional stability, and openness to new experiences were assessed using the Ten Item Personality Inventory (TIPI) (34). The response scale is a 7-point Likert scale ranging from 1 = “disagree strongly” to 5 = “agree strongly.” The convergent validity of this short questionnaire with other, more detailed personality inventories is sufficient. Psychiatric diagnoses were assessed using a structured clinical interview based on DSM-5/ICD-10 criteria by the above-mentioned interviewer. The Mini-diagnostic interview for psychiatric disorders (Mini-DIPS) covers major psychiatric diagnoses including mood and anxiety disorders, substance use disorders and somatoform disorders. It exhibits high interrater reliability (*κ* > 0.80 for most diagnoses) and good convergent validity with other structured interviews (e.g., SCID) (35).

### Statistical analysis

Statistical analyses were performed using SPSS, Version 29.0.0.0. We conducted descriptive analyses to assess sociodemographic characteristics, prevalence rates of mental disorders, working conditions, and experiences of violence. To examine potential sociodemographic differences between the groups of female sex workers and female social workers, Chi^2^-tests for categorial variables and for the continuous variable “age” a Mann–Whitney *U* test for non-normally distributed data were conducted. Due to the large number of categories and low cell counts, the variable “Nationality” was aggregated into three broader regions (German, East European, other) to ensure valid statistical testing. Years of school education were grouped into three categories reflecting no education or low (≤9 years), medium (10–12 years), and high educational attainment (≥13 years). Risk factors for mental illness in FSW were determined using bivariate logistic regression analyses. The dependent variable was the presence of a mental disorder diagnosis, assessed either as current (point prevalence) or lifetime prevalence, based on the Mini-DIPS interview. For each analysis, the outcome variable was binary (0 = no diagnosis, 1 = diagnosis). Predictor variables included sociodemographic factors (e.g., age, nationality, homelessness, income), work-related experiences (e.g., coercion, experiences of threat and control, perceived job stress), and support indicators (e.g., having someone to trust). We excluded variables with more than five missing values and similar items to avoid multicollinearity. Results of the binomial logistic regression models are indicated as Odd’s Ratios (Exp(B)) with 95% confidence intervals (CI) and overall level of significance set to *p* < 0.05. A two-step-cluster analysis was conducted to distinguish different groups within the sex workers’ population, categorizing respondents according to similar characteristics. To examine group differences in the prevalence of mental disorders in the three clusters compared with the control group, Chi^2^-tests of independence were conducted. A global Chi^2^-test was first performed to assess overall differences across all four groups. Subsequently, pairwise comparisons were conducted between each cluster and the control group for relevant disorders. To control for multiple testing, the significance level was adjusted using Bonferroni correction, resulting in an alpha threshold of 0.017.

## Results

### Sociodemographic characteristics

Four hundred three interviews were conducted with sex workers and 157 with the control group of social workers. Two participants subsequently withdrew their consent to participate and their data was excluded from the final evaluation. The age among the sex workers ranged from 18 to 70, with a mean age of 33.8 years (SD 9.2), in the control group the age ranged from 20 to 65 and the mean age was 35.5 years (SD 10.2). 92.5% of the sex workers identified as female, 1% as male and 6.5% as non-binary, while 96.8% of the social workers identified as female and 3.2% as non-binary. Regarding the migration background there was a difference between the groups: While 95.5% of the control group had German citizenship, this was the case for only 58.8% of the sex workers. The rest came predominantly from Eastern European countries (7.5% Bulgarian, 6% Hungarian, 2.5% Polish, 2.5% Romanian, 1.3% Russian, 1.3% Ukrainian, 0.5% Serbian, 0.5% Czech and 0.3% Moldovan), 3.8% was Vietnamese and the rest had other origins. Of the non-German sex workers, 67.4% had a residence permit or visa, while 9.9% lacked permission to stay. In the control group 95.5% had the German citizenship. Clear differences were also evident in educational attainment: 89.8% of the control group had completed a university degree, while only 60.8% of the sex workers had vocational training, 13.2% had no formal qualifications and only 2.5% never attended school. Homelessness was significantly more common among the sex workers: 25.1% reported being currently or previously affected, in contrast to the control group, where no such information was provided. Half of the participants in the sex worker sample (48.9%) reported being in a relationship, with 8.3% stating that their partner was unaware of their profession. In the control group, 68.8% had a steady relationship. While no significant difference between the groups were found in age (*U* = 34,160, *p* = 0.117), gender [*χ*^2^(2) = 3.97, *p* = 0.137] and having children [*χ*^2^(1) = 1.16, *p* = 0.280], significant group differences emerged regarding nationality [*χ*^2^(2) = 15.23, *p* = 0.005], education [*χ*^2^(2) = 9.66, *p* < 0.001], vocational training [*χ*^2^(1) = 6.77, *p* < 0.001], relationship status [*χ*^2^(1) = 10.25, *p* < 0.001], having someone to trust [*χ*^2^(1) = 9.20, *p* < 0.001], age of entry (*U* = 37171.5, *p* < 0.001), and monthly income [*χ*^2^(2) = 12.69, *p* < 0.001]. These variables were taken into account when interpreting subsequent analyses. Table 1 provides detailed comparison of the sociodemographic characteristics of the two groups and Table 2 summarizes the sociodemographic differences between the two study groups based on statistical testing.

**Table 1:** Sociodemographic data of the two groups.

Variable	Sex worker		Control group	
	Percent/mean	*n*	Percent/mean	*n*
Age (mean)	33.8	403	35.5	157
Gender				
Female	92.5	372	96.8	152
Male	1	4	0	0
Non-binary	6.5	26	3.2	5
Nationality				
German	58.8	235	95.5	150
East European	22.4	90	2	3
Other	18.8	78	2.5	4
Residence status		141		
Residence permit	62.4	88		
Limited residence permit	5	7		
No residence permit	9.9	14		
Other	22.7	32		
Education				
Without	13.2	53	0	0
High school	62.4	251	10.2	16
University	22.8	92	89.8	141
Partner (Yes)	48.9	194	68.8	108
Children (Yes)	31.4	126	26.8	42
Someone to trust (Yes)	83.5	334	99.4	156
Entry age (mean)	25.2	397	26.5	156
Income in EUR per month				
None	2.5	10	0	0
Up to 1,000 Euro	36.6	145	5.2	8
More than 1,000 Euro	60.9	241	94.8	149

**Table 2:** Group differences in sociodemographic characteristics between female sex workers and female social workers.

Variable	Test	Test statistic	df	*P*-value
Age	Mann–Whitney U	*U* = 34,160	–	0.117
Gender	Chi^2^-Test	*χ*^2^(2) = 3.97	2	0.137
Nationality	Chi^2^-Test	*χ*^2^(2) = 15.23	2	**0.005**
Education	Chi^2^-Test	*χ*^2^(2) = 9.66	2	**<0.001**
Vocational training	Chi^2^-Test	*χ*^2^(1) = 6.77	1	**<0.001**
Relationship status	Chi^2^-Test	*χ*^2^(1) = 10.25	1	**<0.001**
Children	Chi^2^-Test	*χ*^2^(1) = 1.16	1	0.280
Someone to trust	Chi^2^-Test	*χ*^2^(1) = 9.20	1	**<0.001**
Age of entry	Mann–Whitney *U*	*U* = 37171.5	–	**<0.001**
Monthly income	Chi^2^-Test	*χ*^2^(2) = 12.69	2	**<0.001**

Statistical significance (*p*-value<0.05) is indicated by bold figures.

### Reasons for engaging in sex work or social work

The age at which participants entered employment differed significantly between the groups: While sex workers entered the industry on average at 25.2 years (range: 18–60 years), the average entry age for social workers was 26.5 years. 8.3% of sex workers entered the industry as minors, which was not the case in the control group. The motives for entering the respective professions, also showed considerable differences: Financial reasons were cited as a motive by 71.3% of the FSW, but only by 7% of social workers. Enjoyment of the work was cited by 56.4% of sex workers and significantly more – 92.9% – of social workers. Altruistic reasons, such as supporting family or partner, played a role for 39.5% of sex workers, while only 13.3% of social workers cited this reason. Debt reduction (18.4%) and financing drug use (10.6%) were specific motives in the sex work group that were not or hardly present in the control group. 12.1% of the social workers stated that they had taken up their work to finance their own training – a reason that was also mentioned by 18.4% of the sex workers. A feeling of having “no other choice” was expressed by 10.7% of the sex workers and feeling “forced by circumstances” by 19.4%, while only 1.9% of the social workers reported this. 3.7% of the sex workers reported concrete experiences of coercion – an aspect that was completely absent in the control group as anticipated. Table 3 provides an overview of the motives in the two groups.

**Table 3:** Reasons for engaging in the respective work of the two groups.

Variable	Sex worker		Control group	
	Percent	*n*	Percent	*n*
Financial reasons	71.3	287	7.0	11
Enjoyment	56.4	227	92.9	146
Altruistic reasons	39.5	159	13.3	21
Supporting family	28.6	115	9.5	15
Supporting partner	10.9	44	3.8	6
Dept reduction	18.4	74	6.3	10
Financing drug use	10.6	43	0	0
Financing training	18.4	74	12.1	19
No other choice	10.7	43	0	0
Forced by circumstances	19.4	78	1.9	3
Forced by someone	3.7	15	0	0
Other	17.4	70	29.2	46

### Conditions and work setting

Many FSW reported working in multiple settings and all answers were weighted equally. The majority (52.3%) cited meeting their customers in a hotel, around two thirds offered services in the customer’s flat (30.3%) or in a specially rented flat or studio (29.3%), 11.7% worked in brothels, 8.5% in special clubs, 4.5% in (massage) parlors and 30.3% online. 15.2% said they worked as escorts in different locations and 11.5% met their clients in their private flat (Table 4). A subgroup of women worked in street-based settings (23.5%), in cars (17.7%) or in campers (2.2%). The length of time participants had been engaged in sex work ranged from 1 month to 49 years, with a mean duration of 7.1 years. The majority (60.9%) had a monthly income of more than 1,000 Euro, 36.6% earned less and 2.5% reported having no available income.

**Table 4:** Work setting, motivation and experiences of FSW.

Variable	Percent	*n*
Work setting		
Own flat	11.53	46
Rented flat/Studio	29.32	117
Brothel	11.78	47
(Massage)salon	4.51	18
Club	8.52	34
Client’s flat	30.33	121
Car	17.79	71
Hotel	52.38	209
Street	23.56	94
Camper	2.26	9
Online	30.33	121
Other	19.30	77
Escort	15.29	61
Desire to leave (Yes)	39.6	157
Reasons for doing the job		
Enjoying it	56.47	227
Financial reasons	71.39	287
Supporting family	28.61	115
Supporting partner	10.95	44
Paying off depts	18.41	74
Financing drugs	10.70	43
Financing education	18.41	74
No other choice	10.70	43
Coerced by someone	3.73	15
Forced by circumstances	19.40	78
Other	17.41	70
Coerced into job (Yes)	10.9	44
Experience of violence		
Sexual	10.4	41
Physical	12.3	49
Threatened	17	68
Experience with customers		
Good	60.8	243
Neither nor	27.1	108
Bad	12	48
Well-being		
Good	63.3	254
Neither nor	18.7	75
Bad	18	72
Duration (mean)	7.1	376

### Experience of violence and coercion

Among the sex workers two third of the respondents (60.8%) described their experiences with customers as mostly good, 27.1% reported a neutral relationship with their clients and only 12% stated mostly negative experiences. 63.3% reported job satisfaction and well-being, 18% stated they felt bad about their work, and 39.6% expressed a desire to leave the industry. Several women experienced violence in the context of their job: 10.9% said they were initially coerced to do this job, 10.4% had faced sexual violence in the past 6 months, 12.3% physical violence and 17% felt threatened or controlled.

### Mental health outcomes in FSW

Point prevalence and lifetime prevalence rates of mental disorders were assessed via diagnostic interviews. Table 5 provides a detailed breakdown of the psychiatric diagnoses among female sex workers and the control group. Logistic regression analyses were conducted to identify correlates of the point prevalence of mental disorders among FSW in order to explore group-specific risk factors. The analyses focused on identifying intra-group predictors. In contrast, since the social worker group served primarily as a comparison sample to contextualize mental health outcomes, a multivariate analysis of correlates was not pursued in the same depth. The following variables were considered statistically significant at *p* < 0.05 and are therefore significant correlates of mental health outcomes within the sex worker sample: homelessness (OR = 0.11, CI = 0.02–0.48, *p* = 0.003; Ref = yes): Compared to homeless FSW (reference group), non-homeless FSW had significantly lower odds of experiencing a mental disorder, indicating that homelessness is associated with increased risk of mental illness. Monthly income (OR = 0.50, CI = 0.25–0.97, *p* = 0.042; Ref < €1,000): Compared to FSW with lower income (reference group), those earning ≥ €1,000 per month had significantly lower odds of experiencing a mental disorder, suggesting a protective effect of higher income. Perceived job stress (OR = 2.83, CI = 1.11–7.18, *p* = 0.029; Ref = low): FSW reporting higher job stress had significantly higher odds of experiencing a mental disorder. Experiences of threat and control (OR = 0.15, CI = 0.03–0.84, *p* = 0.030; Ref = no): Those who had not experienced threat and control (reference group) had significantly lower odds, indicating that such experiences are risk factors (Table 6). For lifetime prevalence, residence status was a significant correlate (OR = 1.33, CI = 1.05–1.69, *p* = 0.018; Ref = no), with those holding less secure residence status showing higher odds of mental disorder. All results were interpreted in reference to the coding of each variable. Age and other continuous variables (e.g., nationality, years of education, entry age into sex work) were treated as continuous predictors in the logistic regression model.

**Table 5:** Point prevalence and lifetime prevalence rates for different psychiatric disorders in the two groups.

Variable	Point prevalence (%)		Lifetime prevalence (%)	
	Sex workers	Social workers	Sex workers	Social workers
All disorders	60.3	44.2	77.9	56.3
Anxiety disorders	35.5	28.7	36.1	31.0
Panic disorder	18	7.7	30.7	23.7
Generalized Anxiety	14.5	9	16.7	12.8
Agoraphobia	10	3.8	14	7.7
Social phobia	10.7	6.4	13	11.5
Specific phobia	10.7	12.2	11.2	12.8
Obsessive Compulsive disorder	6.5	1.9	10	4.5
Affective disorders	24.3	10.2	54.7	45.0
Major depression	20.9	9.6	49.6	43.6
(Hypo)Mania	7	1.3	16.5	4.5
Dysthymia	9.5	5.1	14	10.3
PTSD	17	1.9	33.9	21.8
Acute stress disorder	0.7	0	1	0
Eating disorders	8.2	3.2	24.2	23.1
Somatoform disorders	3.7	4.5	4.7	5.1
Sleep disorders	18.2	14.8	20.9	18.6
Substance use disorders	19.7	0.6	27.7	4.4
Gambling disorder	2.7	0	4.7	0
Schizophrenia	2	0.6	6.2	1.2

**Table 6:** Bivariate logistic regressions for different variables.

Variable	OR (95% CI)	*P*-value
Age (continuous)	0.97 (0.89–1.05)	0.540
Nationality (continuous)	1.01 (0.96–1.06)	0.610
Residence permit (ref = no)	1.01 (0.82–1.24)	0.897
Partner (ref = no partner)	0.85 (0.26–2.80)	0.799
Years of education (continuous)	1.04 (0.87–1.25)	0.611
Vocational training (ref = no)	0.57 (0.17–1.90)	0.364
Homelessness (ref = yes)	0.11 (0.02–0.48)	**0.003**
Entry age (continuous)	1.01 (0.92–1.11)	0.701
Monthly income (ref < €1,000)	0.50 (0.25–0.97)	**0.042**
Workplace (ref = indoor)	0.84 (0.68–1.04)	0.124
Someone to trust (ref = no)	0.20 (0.03–1.14)	0.071
Well-being (continuous score)	0.71 (0.39–1.29)	0.273
Negative experience with customers (ref = none)	0.87 (0.59–1.28)	0.479
Coerced into job (ref = no)	0.54 (0.07–3.91)	0.542
Experience of violence in the last 6 months		
Sexual (ref = no)	0.92 (0.10–8.31)	0.946
Physical (ref = no)	0.91 (0.15–5.62)	0.926
Threat/control (ref = no)	0.15 (0.03–0.84)	**0.030**
Perceived burden		
Caused by job (ref = low)	2.83 (1.11–7.18)	**0.029**
Caused by coercion (ref = low)	0.40 (0.14–1.14)	0.087
Caused by violence (ref = low)	2.08 (0.80–5.36)	0.130
Desire to leave (ref = no)	0.71 (0.18–2.77)	0.628

Current diagnosis of psychiatric disorder assessed by Mini-DIPS (dependent variable) in the group of FSW. Statistical significance (*P*-value < 0.05) is indicated by bold figures.

### Cluster analysis among FSW

**Figure 1:**
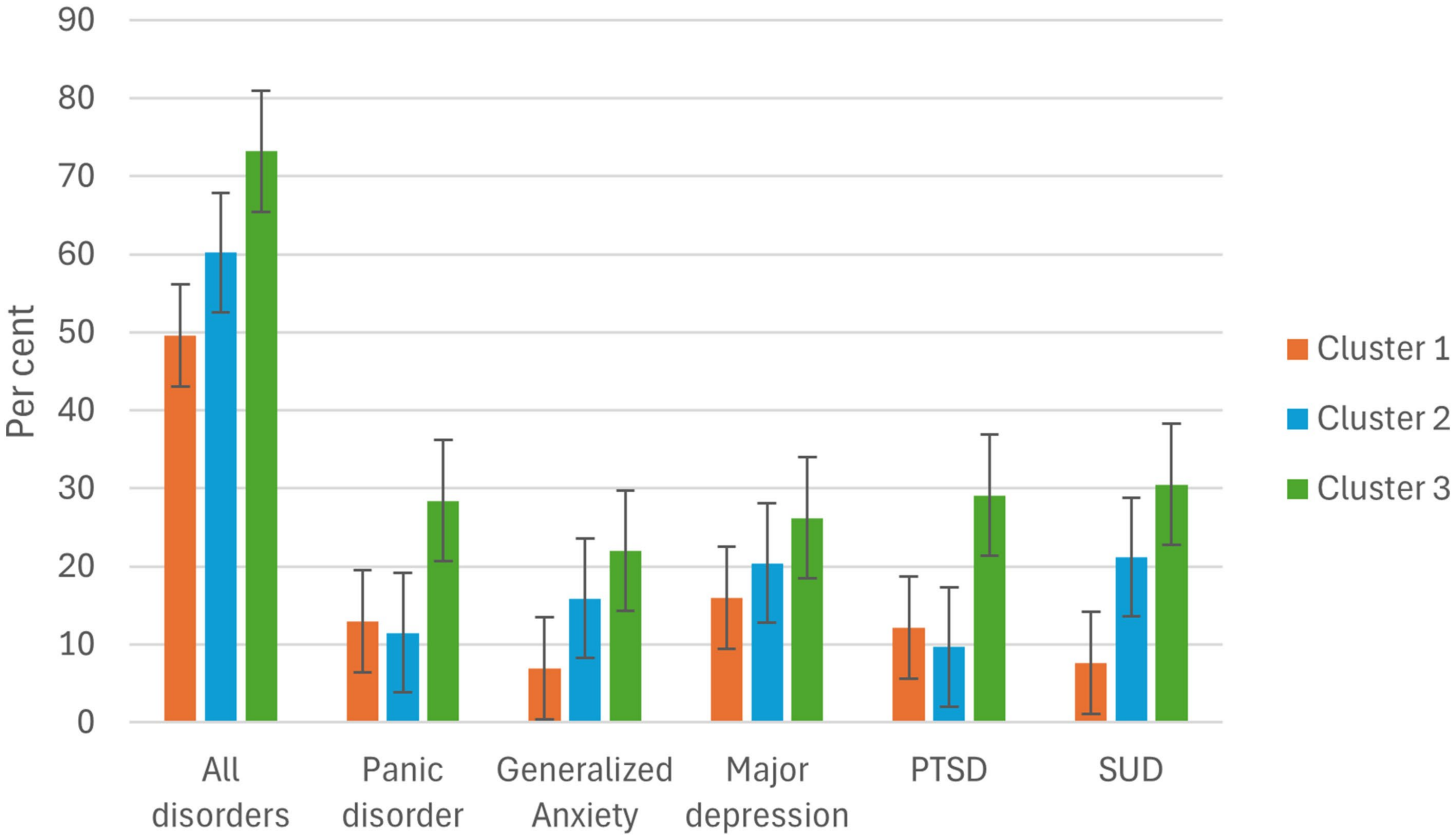
Percent of point prevalence rates in different groups of FSW.

A two-step cluster analysis was performed using explanatory variables derived from the logistic regression results and guided by theoretical considerations and interpretability. The variable “monthly income” was selected to reflect precarious living conditions, as it was both statistically significant and conceptually relevant to economic vulnerability. Although “years of education” and “having someone of trust” were not statistically significant in the bivariate analyses, they were included due to their theoretical relevance as potential indicators of resilience. The variable “experience of threat and control” was selected over “experiences of sexual or physical violence” not only because it showed statistical significance, but also because it captures a broader and ongoing form of coercion that may be more relevant in the context of structural vulnerability. Variables representing broader constructs such as stigmatization were excluded to avoid redundancy and ensure clearer cluster interpretation. Instead, the variable “perceived burden on the job” was used as a proxy for self-stigma, aligning with qualitative findings and theoretical models on internalized stigma. Based on the Bayesian Information Criterion (BIC), a three-cluster solution was determined to be the most parsimonious. The first cluster (*n* = 131) includes mainly FSW with higher education, a monthly income of over 1,000 EUR and reported low levels of violence related to their work and no work-related stress. The second cluster (*n* = 113) consists of women working for a monthly income of up to 1,000 EUR. Education of FSW in this group and experiences on the job vary. The third cluster (*n* = 142) includes sex workers with low education who mainly work for an unstable monthly salary and experience the highest job stress. This group reports the highest rates of violence. The cluster analysis identified meaningful subgroups within the sample of FSW. Cluster 1, which showed more favorable indicators such as higher income and education, was used as a reference group. Compared to this group, women in cluster 3 reported significantly more experiences of threat and control and lower income, indicating higher vulnerability. Figure 1 shows the distribution of point prevalence rates in the three clusters.

The three clusters show considerable differences in mental health outcomes. Cluster 3 has the highest prevalence rate of mental disorders (73.2%), including substance abuse (30.5%), panic disorder (28.4%), generalized anxiety disorder (GAD) (22.0%), depression (26.2%) and PTSD (29.1%). Depression (20.4%) and SUD are also very common in cluster 2 (21.2%), which includes FSW who have been homeless at some point in their lives. In contrast, cluster 1 represents a relatively healthy subgroup, with depression being the most common disorder (16.0%).

### Comparisons with the control group

Chi^2^-tests revealed significant differences between the groups (Table 7). Results were considered statistically significant at *p* < 0.05, so that all of the following variables meet this criterion. Regarding anxiety disorders prevalence rates of panic disorder [*χ*^2^(3) = 12.89, *p* = 0.005] and agoraphobia [*χ*^2^(3) = 12.10, *p* = 0.007] were significantly higher among the FSW compared to the social workers and for affective disorders significantly higher prevalences were found for depression [*χ*^2^(3) = 10.05, *p* = 0.018], (hypo)mania [*χ*^2^(3) = 28.00, *p* < 0.001] and dysthymia [*χ*^2^(3) = 14.75, *p* = 0.002]. Regarding stress-related disorders prevalences of PTSD [*χ*^2^(3) = 22.83, *p* < 0.001] and acute stress disorder [*χ*^2^(3) = 11.30, *p* = 0.010] were significantly higher among FSW. Also, significantly higher prevalences were found for substance use disorders (SUD) [*χ*^2^(3) = 52.55, *p* < 0.001], Gambling disorder [*χ*^2^(3) = 8.67, *p* = 0.045] and Schizophrenia [*χ*^2^(3) = 8.67, *p* = 0.034]. For the most diagnoses we found significantly higher rates in the study group of FSW compared to the control group, in particular the difference in PTSD prevalences was noticeable: 17–1.9%. An exception represented somatoform disorders, which were more frequent among social workers (4.5%, compared to 3.7%).

**Table 7:** Differences of point prevalence rates between FSW and the control group.

Variable	*X*^2^(3)	*P*-value
All disorders	16.16	**<0.001**
Anxiety disorders		
Panic disorder	12.89	**0.005**
Generalized Anxiety	6.47	0.091
Agoraphobia	12.10	**0.007**
Social phobia	5.54	0.136
Specific phobia	5.43	0.143
Obsessive Compulsive disorder	6.47	0.091
Affective disorders		
Major depression	10.05	**0.018**
(Hypo)Mania	28.00	**<0.001**
Dysthymia	14.75	**0.002**
PTSD	22.83	**<0.001**
Acute stress disorder	11.30	**0.010**
Eating disorders		
Anorexia	3.68	0.299
Binge eating disorder	5.70	0.127
Somatoform disorders	7.20	0.066
Sleep disorders	0.98	0.806
Substance use disorders	52.55	**<0.001**
Gambling disorder	8.76	**0.045**
Schizophrenia	8.67	**0.034**

Statistical significance (*P*-value<0.05) is indicated by bold figures.

Comparing the three clusters of FSW with the control group, a global chi-square test indicated that the prevalence of mental disorders differed significantly between the four groups (*χ*^2^ = 31.196, df = 12, *p* = 0.002). To identify where these differences occurred, post-hoc chi-square tests were conducted comparing each of the three clusters individually to the control group. Bonferroni correction was applied for the three comparisons per disorder, setting the adjusted significance threshold at *α* = 0.017. The prevalence of GAD was significantly higher in clusters 2 and 3 compared to the control group (*χ*^2^ = 9.14, *p* = 0.002; *χ*^2^ = 8.63, *p* = 0.003). MD was significantly more prevalent in cluster 3 than in the control group (*χ*^2^ = 7.40, *p* = 0.007). For SUD, significant differences were observed for cluster 2 (*χ*^2^ = 8.48, *p* = 0.004) and cluster 3 (*χ*^2^ = 8.01, *p* = 0.005). No significant differences were found in panic disorder or PTSD after correction for multiple comparisons. An overview of the chi-square results is presented in Table 8.

**Table 8:** Chi-square comparisons between each cluster and the control group for point prevalences.

Diagnosis	Group comparison	*X*^2^(1)	*P*-value	Significant (*α* = 0.017)
Panic disorder	Cluster 1 vs. Control group	1.614	0.204	No
Panic disorder	Cluster 2 vs. Control group	0.001	0.980	No
Panic disorder	Cluster 3 vs. Control group	1.188	0.276	No
GAD	Cluster 1 vs. Control group	9.142	0.019	No
GAD	Cluster 2 vs. Control group	5.489	**0.002**	Yes
GAD	Cluster 3 vs. Control group	8.634	**0.003**	Yes
MD	Cluster 1 vs. Control group	1.307	0.253	No
MD	Cluster 2 vs. Control group	3.745	0.053	No
MD	Cluster 3 vs. Control group	7.400	**0.007**	Yes
PTSD	Cluster 1 vs. Control group	3.464	0.063	No
PTSD	Cluster 2 vs. Control group	3.693	0.055	No
PTSD	Cluster 3 vs. Control group	3.715	0.054	No
SUD	Cluster 1 vs. Control group	3.428	0.064	No
SUD	Cluster 2 vs. Control group	8.476	**0.004**	Yes
SUD	Cluster 3 vs. Control group	8.010	**0.005**	Yes

Statistical significance (*α* = 0.017) is indicated by bold figures.

### Comparisons with the general population

Point prevalences for anxiety disorders in the study sample were 35.5% which is more than twice as high than in the general population (15.4%). Most frequent among sex workers were panic disorders (18%) and GAD (14.5%), prevalence of other anxiety disorders (agoraphobia, social phobia and specific phobia) were 10%. Affective disorders showed prevalences of 24.3% with depression 20.9%, more than two times higher than in the general population (9.8%, depression: 8.2%). SUD were the second most common after depression with point prevalences of 19.7% more than three times higher than in the general population (5.7%) (22).

**Figure 2:**
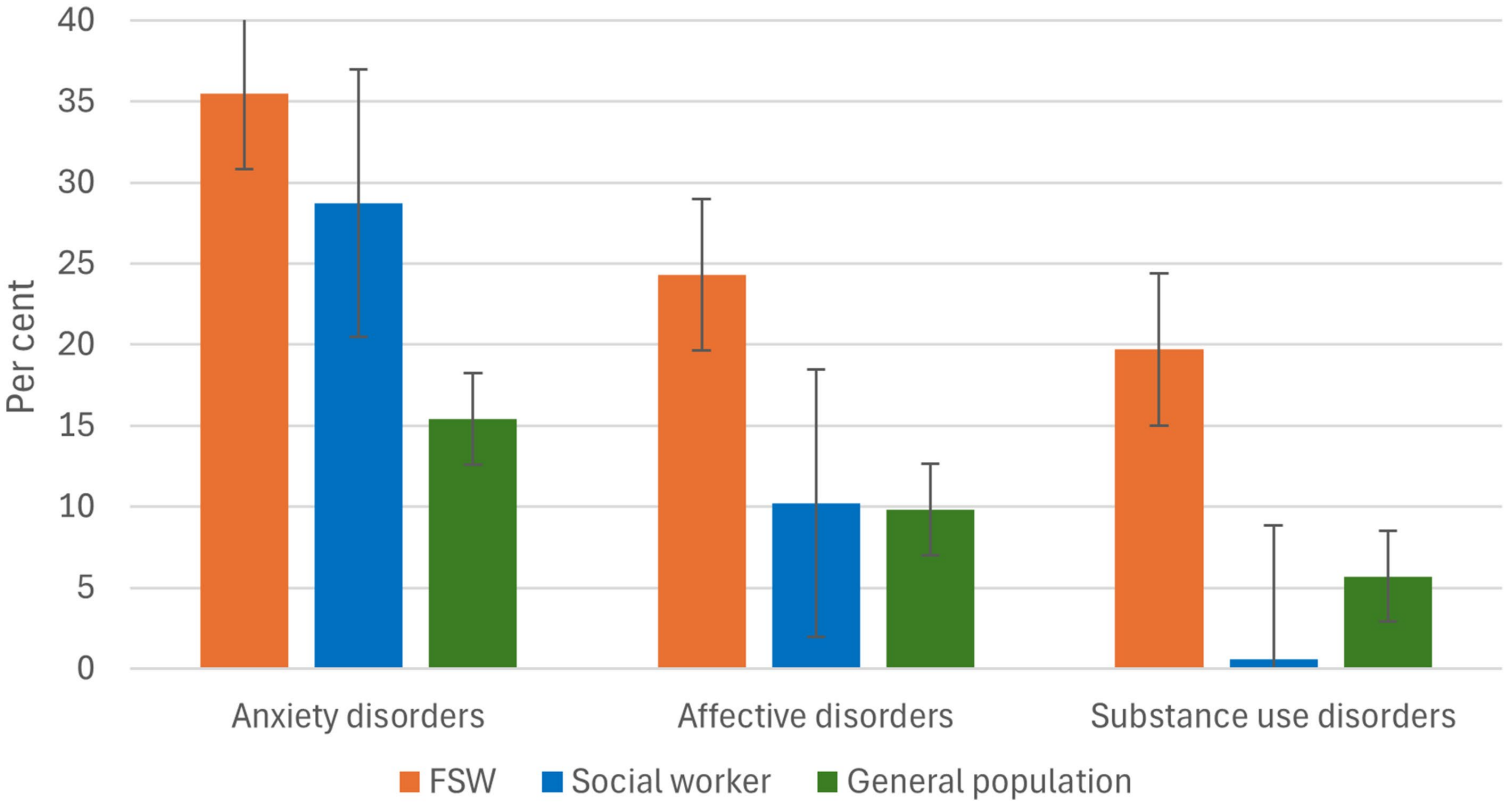
Point prevalences of relevant disorders among FSW, social workers and the general population.

The control group of social workers also had higher prevalence rates of anxiety disorders than in the general population (28.7%) as for affective disorders (10.2%). Most frequent in this group were specific phobias (12.2%) and GAD (9.0%) as well as depression among affective disorders (9.6%). Substance-related disorders were less represented with 0.6%. The same applies for stress-related disorders (PTSD: 1.9%), eating disorders (3.2%) and schizophrenia (0.6%) which were represented more often in the sex worker sample. Figure 2 provides an overview of relevant prevalence rates in the two experimental groups compared to the general population.

Lifetime prevalence rates of various mental disorders in the German general population vary depending on the study and methodology. Substance-related disorders represent the most common group with a lifetime prevalence of 25.8%. Anxiety disorders affect around 15.1% of the population over the course of their lives. The lifetime prevalence rate of affective disorders (including depression) is 12.3%. As for PTSD estimates vary between 1.5 and 2% in the general population. For eating disorders, the lifetime prevalence is given as 0.7%. Regarding sleep disorders, current specific lifetime prevalence data are limited. However, in 2023, around 7.3% of insured persons received a diagnosis of a sleep disorder, which indicates an increase compared to 5.5% in the last 10 years (23).

Lifetime prevalences in the current study were higher for anxiety and affective disorders, PTSD, eating and sleep disorders in both groups among FSW and social workers. Substance related disorders among sex workers occurred comparably to prevalence rates in the general population (27.7%).

## Discussion

The aim of this study was to investigate risk and resilience factors for mental health of FSW and compare them with a control group of social workers. Central research questions regarding psychological distress and symptoms in this population, the distribution of risk and resilience factors, and differences between FSW and a professional group outside of sex work were addressed. The results based on these questions are discussed below. Both theoretical and practical implications are discussed.

### High prevalences of mental disorders among FSW

Corresponding to previous research, FSW exhibited an increased prevalence of psychological symptoms. Since anxiety disorders, affective disorders and SUD are among the most frequent mental disorders in Germany (22), high prevalence rates of these disorders were not unusual in the samples studied. Compliant to results in previous studies (21), point prevalence rates of those disorders were higher than those in the general population but also in the control group. Key risk factors in the sex worker group were experienced psychological violence (e.g., threat and control), economic insecurity (e.g., homelessness, low-income status) and internalized stigma (perceived work-related stress). The experience of structural insecurity as well as psychological burden had similar stressful impact on mental health. These aspects are consistent with the findings of Rössler et al. (21), highlighting structural and psychological violence as main risk factors in the sex work sector. On the other hand, resilience factors also became apparent. These included, in particular, social support in the private environment, professional self-determination and the ability to set boundaries. Interestingly, several participants described a high degree of professional self-confidence and control over their actions, which differentiates the common victim narrative. It should be also emphasized that not all of the FSW interviewed were under severe psychological stress. What must be taken into account, however, is the diversity of experiences against the background of different educational levels and structural privileges. FSW represent a heterogeneous group with varying characteristics, since age, background and education span a wide range. The within-group comparison of the cluster analysis highlights the heterogeneity among FSW and points to the need for differentiated intervention strategies based on varying levels of structural and psychosocial risk factors. *Post hoc* analyses revealed significantly higher prevalence of GAD in two of the three clusters compared to the control group. This may reflect a heightened level of chronic stress and anxiety symptoms in the sex worker sample. Cluster 3, which appeared to represent the most psychologically burdened subgroup, also showed significantly higher rates of major depression and SUD compared to the control group. The lack of significant differences in panic disorder and PTSD, although expected, may be due to statistical limitations.

What should be noted as well is that the most vulnerable group, such as women trafficked into sex work, are significantly underrepresented due to the difficulty of reaching them. However, not all participants interviewed entered the industry by choice. Around 10% were initially coerced into sex work, others felt constantly forced by someone (3.7%) or by the circumstances (19.4%). Many felt threatened or controlled (17%) or became economically dependent due to debt (18.4%). These are circumstances that cannot be ignored. However, for some, sex work represents a sense of autonomy, self-esteem, or empowerment (28).

### Key findings in the control group

Significant differences in the mental health of FSW emerged compared to the control group of social workers: While experiences of stress were also described in the control group, clinically relevant symptom clusters occurred significantly less frequently and with less intensity. This suggests that specific stressors in the context of sex work (such as violence, structural insecurity, psychological stigma) play a central role in mental health. However, social workers still had higher rates of mental health problems than the general population (e.g., anxiety disorders: 28.7% vs.15%; affective disorders: 10.2% vs. 9.8%). Possible explanations include the emotional intensity of care work, high workload and chronic exposure to stress, moral injuries and systematic constraints, as well as occupational gender dynamics. While these rates were still significantly lower than among sex workers, they are notable and underscore how care work, even in professionalized contexts, still incurs significant psychological cost.

Participants in the control group were predominantly highly educated, had secure legal status, and stable living conditions as protective factors for mental health. The majority (92.9%) stated that they enjoyed their job, suggesting higher intrinsic motivation and greater occupational identification, in contrast to FSW, where economic motives and feelings of compulsion were more frequent. Female social workers more frequently cited organizational resilience factors (e.g., structured working conditions), whereas resilience among FSW was strongly developed individually. This suggests different mechanisms of coping strategies, which may be partly due to the structural embedding of the profession. As expected, the two groups differed significantly in certain sociodemographic characteristics, such as nationality, education and vocational training, relationship status, having someone to trust and monthly income. These differences likely reflect broader structural inequalities and should be considered when interpreting the observed differences in mental health outcomes. However, since no significant differences were found in other variables such as age and gender, it is unlikely that the observed disparities in mental health are solely due to sociodemographic factors. Rather, they appear to be closely linked to the distinct life circumstances of the two groups.

### Recommendations

In the present study, insecure residency status, a history of homelessness, and unstable monthly income correlated with mental health problems. These results are consistent with previous findings showing that financial instability is an important predictor of poor mental health among sex workers (36). Furthermore, the prevalence rates of mental health disorders were highest in the group of FSW with unstable monthly income and frequent work stress. This demonstrates that economic stability is essential to reduce mental health risks and ensure access to adequate healthcare. Affordable health insurance solutions, such as social security funds, are conceivable, and anti-price gouging measures, such as fair pricing agreements, could help alleviate financial pressure. Furthermore, mobile health services can provide proactive medical care directly at the workplace, ensuring access for those who may not be able to access traditional healthcare services due to homelessness and lack of a residence permit (37).

As some of the interviewed FSW felt burdened by their job, another critical factor is the internalized stigma, which contributes to low self-esteem and psychological distress (38, 39). The impact of internalized stigma on psychological distress in marginalized and vulnerable groups is addressed in various studies showing that it negatively affects access to treatment and support as well as it exacerbates poor mental health outcomes. Perceived, experienced and anticipated stigma was assessed in the interviews and nearly 20% of FSW in our study reported feeling bad about their work, showing that work-related self-stigma was also a strong predictor of mental illness. Strengthening the community through empowerment programs and mentoring initiatives, could provide sex workers with self-advocacy and support (14). Encouraging self-determined work models could promote autonomy and independence, while decriminalizing sex work and abolishing compulsory registration could help reduce stigma and remove legal barriers (9).

In our study, women exhibited high rates of mental health diagnoses such as substance use. The additional stigmatization of mental illness and certain aspects of substance use, along with both internalized and institutional stigma faced by sex workers, may further restrict opportunities to address needs in health care services. Medical professionals, including doctors and therapists, should receive specialized training to increase awareness and sensitivity to issues like discrimination, fear of stigmatization, unclear residence status or lack of health insurance (7, 9, 14). Dedicated psychotherapeutic services should be established in hospitals and outpatient clinics to provide tailored mental health support (29).

Since social workers, who often work with sex workers in outreach, mental health, or advocacy settings, also suffered from psychological distress, expanding counseling and support services through policy changes is essential to improve the situation for both groups. Increased funding and professional training for counseling centers would improve their ability to provide psychological, legal, and social support (40–42). Increased collaboration between counseling centers, authorities, and interdisciplinary teams would improve the coordination of available resources (43).

These comprehensive measures aim to create a safer, more supportive, and inclusive environment for sex workers. Alternative legal models such as the “Nordic Model,” which criminalizes the purchase of sexual services but not their sale, are often touted as protecting sex workers. However, evidence suggests that such approaches continue to contribute to stigma, policing, and unsafe working conditions, ultimately affecting mental health and access to services (44–46). Given the links identified in this study between structural vulnerabilities (e.g., homelessness, insecure residency status) and mental health, decriminalization models that prioritize harm reduction and labor rights may better promote the well-being of sex workers. Further research is needed into the type of psychological treatment that would be most effective for FSW, testing brief psychological interventions through existing outreach services. Addressing their medical, psychological, economic and social needs is essential to fostering well-being and societal acceptance. By implementing these strategies, policymakers and service providers can contribute to reducing health disparities and improving the overall quality of life for individuals engaged in sex work.

### Strengths and limitations

So far, this study represents the largest quantitative assessment of female sex workers’ mental health across various work settings in Germany. Due to the open, mobile, and loosely defined nature of this population, obtaining a random sample was not feasible. However, the chosen sampling method approximates a representative sample as closely as possible. A strength of this study is the large sample of FSW, which enabled the identification and analysis of distinct subgroups. This level of detail, rarely achieved in this field due to the typically small sample size, provides valuable insights into specific sex work contexts.

A limitation of this study is the potential for sampling bias, as participation was based on individuals’ personal motivations. Furthermore, the study does not establish causality – it remains unclear whether mental health disorders precede sex work or result from it. Another limitation was our inability to include women, who did not speak any of the interview languages. Counseling centers, e.g., often work with sex workers from Latin America, Africa and Asia who could not participate if they did not speak one of our offered interview languages. Legal constraints also precluded the inclusion of women under 18 years of age. Hence, since none of the participants were minors, the findings apply solely to adult FSW.

Furthermore, the study did not capture data from women who are victims of forced prostitution. Although we were able to interview women who felt forced into work by (ex-)partners or others, we were unable to reach women who are currently forced into prostitution under the influence of procurers or human trafficking, meaning that the prevalence of violence experienced by some respondents may be underestimated. Consequently, the study is not fully representative, as the participant group does not precisely reflect the overall composition of the FSW population. Nonetheless, the inclusion of a diverse range of sex workers provides a valuable overview of sex work across various settings and the associated challenges. The findings clearly indicate a strong relationship between the mental health of sex workers and their working conditions.

The use of interviews for data collection may have led to biases such as interviewer bias, social desirability, and inconsistency. However, to obtain detailed, contextualized accounts of experiences with mental distress, this format was the best choice. The interviews enabled the building of trust, the explanation of complex topics, and supported participants in potentially distressing disclosures. This method is particularly suitable for marginalized populations exposed to stigma and legal vulnerability, since trust, safety, and a deep understanding are essential for ethical and meaningful research.

The choose of female social workers as control group might be unfavorable since social workers are not demographically or psychologically representative of the general population. They are often higher educated, trained in mental health awareness and more likely to have access to healthcare and social support. This limits generalizability and differences observed may reflect the specific characteristics of social workers rather than the general public. A selection bias could occur with individuals who are emotionally resilient or have certain personality traits being more likely to remain in social work especially given its high burnout risk. This could underestimate the true mental health burden in comparable helping professions. While social workers were a pragmatic and ethically defensible choice for a control group, the comparison might not display the real gap in mental health outcomes between sex workers and other low-threshold or economically marginalized populations. Future research should include multiple or better comparison groups, e.g., nurses or unemployed individuals and balance covariates by statistical adjustments.

## Conclusion

This study assessed the mental health among female sex workers in Germany, comparing them to a control group of social workers. Confirmatory evidence was found for higher prevalence rates in FSW depending on the working conditions, housing situation and experiences on the job.

The fact that residence status, homelessness and an unsteady income are risk factors for mental health is of particular interest and highlights the need of comprehensive measures to improve support services for women engaged in sex work.

## Data Availability

The datasets presented in this article are not readily available because of the sensitive nature of the data. They can be accessed upon request from the authors and with appropriate ethical approval. Requests to access the datasets should be directed to meryam.schouler-ocak@charite.de.
