# Correction: A quantitative study on female sex workers' mental health in Germany

**DOI:** 10.3389/fpubh.2026.1777357

**Published:** 2026-01-27

**Authors:** Franziska Kroehn-Liedtke, Olivia Kalinowski, Gizem Kaya, Anastasiia Lotysh, Hristiana Mihaylova, Krisztina Sipos, Annika Strunk, Lena Zerbe, Wulf Rössler, Meryam Schouler-Ocak

**Affiliations:** Department of Psychiatry and Neurosciences, Psychiatric University Clinic of Charité at St. Hedwig Hospital, Berlin, Germany

**Keywords:** mental health, sex work, prevalence of mental disorders, risk factors, social workers

There was a mistake in Table 1 as published. Instead of 60.9%, a monthly income of more than €1,000 among sex workers was mistakenly reported as 6.9%. The corrected Table 1 appears below.

**Table 1:** Sociodemographic data of the two groups.

**Variable**	**Sexworker**		**Control group**	
	**Percent/ mean**	***n***	**Percent/ mean**	***n***
Age (mean)	33.8	403	35.5	157
**Gender**				
Female	92.5	372	96.8	152
Male	1	4	0	0
Non-binary	6.5	26	3.2	5
**Nationality**				
German	58.8	235	95.5	150
East European	22.4	90	2	3
Other	18.8	78	2.5	4
**Residence status**		141		
Residence permit	62.4	88		
Limited residence permit	5	7		
No residence permit	9.9	14		
Other	22.7	32		
**Education**				
Without	13.2	53	0	0
High school	62.4	251	10.2	16
University	22.8	92	89.8	141
Partner (Yes)	48.9	194	68.8	108
Children (Yes)	31.4	126	26.8	42
Someone to trust (Yes)	83.5	334	99.4	156
Entry age (mean)	25.2	397	26.5	156
**Income in EUR per month**				
None	2.5	10	0	0
Up to 1,000 Euro	36.6	145	5.2	8
More than 1,000 Euro	60.9	241	94.8	149

The original version of this article has been updated.

